# Differential *var* gene transcription in *Plasmodium falciparum* isolates from patients with cerebral malaria compared to hyperparasitaemia

**DOI:** 10.1016/j.molbiopara.2006.08.005

**Published:** 2006-12

**Authors:** Helen M. Kyriacou, Graham N. Stone, Richard J. Challis, Ahmed Raza, Kirsten E. Lyke, Mahamadou A. Thera, Abdoulaye K. Koné, Ogobara K. Doumbo, Christopher V. Plowe, J. Alexandra Rowe

**Affiliations:** a Institute of Immunology and Infection Research, School of Biological Sciences, University of Edinburgh, Edinburgh EH9 3JT, UK; b Institute of Evolutionary Biology, School of Biological Sciences, University of Edinburgh, Edinburgh EH9 3JT, UK; c University of Maryland School of Medicine, Baltimore, MD 21201, USA; d Malaria Research and Training Centre, Faculty of Medicine, Pharmacy and Dentistry, University of Bamako, Bamako, BP 1805, Mali

**Keywords:** DBL, Duffy binding like, PfEMP1, *P. falciparum* erythrocyte membrane protein one, *Plasmodium falciparum*, Virulence, Pathogenesis, Cerebral malaria, Hyperparasitaemia, PfEMP1

## Abstract

The *Plasmodium falciparum* variant erythrocyte surface antigens known as PfEMP1, encoded by the *var* gene family, are thought to play a crucial role in malaria pathogenesis because they mediate adhesion to host cells and immuno-modulation. *Var* genes have been divided into three major groups (A, B and C) and two intermediate groups (B/A and B/C) on the basis of their genomic location and upstream sequence. We analysed expressed sequence tags of the *var* gene DBLα domain to investigate *var* gene transcription in relation to disease severity in Malian children. We found that *P. falciparum* isolates from children with cerebral malaria (unrousable coma) predominantly transcribe *var* genes with DBLα1-like domains that are characteristic of Group A or B/A *var* genes. In contrast, isolates from children with equally high parasite burdens but no symptoms or signs of severe malaria (hyperparasitaemia patients) predominantly transcribe *var* genes with DBLα0-like domains that are characteristic of the B and C-related *var* gene groups. These results suggest that *var* genes with DBLα1-like domains (Group A or B/A) may be implicated in the pathogenesis of cerebral malaria, while *var* genes with DBLα0-like domains promote less virulent malaria infections.

## 1 Introduction

*Plasmodium falciparum* malaria continues to exact a huge toll on health and life throughout the tropics, predominantly among young children in sub-Saharan Africa [1]. The clinical manifestations of falciparum malaria differ markedly from infection to infection; disease symptoms often remain mild, but in some cases severe complications such as cerebral malaria, severe malarial anaemia or respiratory distress develop [2]. The reasons why certain children develop life-threatening complications, while others are able to tolerate very high parasite burdens without severe clinical symptoms remain unclear [3].

Severe malaria has previously been associated with expression of a restricted and antigenically conserved subset of variant erythrocyte surface antigens [4,5], suggesting that expression of certain surface molecules may be associated with specific disease manifestations. The major candidate molecules are the PfEMP1 family (*Plasmodium falciparum* erythrocyte membrane protein one) encoded by the *var* genes [6,7]. PfEMP1 is thought to play a crucial role in host–parasite interaction because it mediates adhesion to host cells to bring about sequestration and immuno-modulation [3]. Each parasite has a repertoire of 50–60 *var* genes [8] and variant surface antigen expression is regularly switched to avoid host recognition [9]. The extracellular portion of PfEMP1 variants contains between two and seven cysteine-rich domains known as Duffy-binding-like (DBL) domains [8]. DBL domains have been classified into five types, α–ɛ, on the basis of consensus motifs, plus a sixth heterogeneous group, DBL-X [8,10]. The PfEMP1 DBLα domain occurs towards the N-terminus of the protein and is the most well-conserved DBL domain. Two distinct types of DBLα domain have been described (α and α1), which differ in the number of conserved cysteines and other hydrophobic residues [11]. For clarification purposes we have here named the two distinct types of DBLα domain as DBLα0 and DBLα1.

On the basis of the full genomic sequence of the 3D7 *P. falciparum* clone, *var* genes have been classified into three major groups (A, B and C) and two intermediate groups (B/A and B/C) defined by the presence of one of three conserved 5′ upstream sequences (UpsA, UpsB or UpsC), and by the gene's position and orientation within a chromosome (summarised in Table 1) [8,12,13]. The Group A and B/A genes are larger and have a more complex domain structure than the other groups [12]. All of the Group A genes have a DBLα1 type N-terminal domain, and two out of four B/A genes have a hybrid DBLα domain with some DBLα1-like features including the lower number of conserved cysteine residues [11]. All of the 3D7 Group B, B/C and C *var* genes have DBLα0 N-terminal domains [11,12]. Amplification of expressed DBLα sequence tags using degenerate primers [14] gives approximately 300–400 base pairs of sequence that can be used to assign expressed *var* genes to either DBLα1-like sequences (Group A or B/A) or DBLα0-like sequences (Group B, C, B/C or B/A) on the basis of the number of conserved cysteine residues in the amplified tag sequence (Table 1).

It has been suggested that the *var* gene groups may recombine separately [12,13], and there is some evidence for different functional properties between groups. Binding to the endothelial receptor CD36 is a property of Group B, B/C and C genes but not Group A genes [11], whereas binding to uninfected erythrocytes to mediate rosetting (a virulence-associated phenotype) [15,16] is a property of the DBLα1 domain of some Group A genes [17,18]. A recent experiment showed up-regulation of some Group A *var* genes in a *P. falciparum* laboratory clone selected using semi-immune serum to express variant surface antigens characteristic of severe malaria [19]. Taken together these studies raise the hypothesis that parasite virulence and clinical disease severity in malaria patients could be related to transcription of particular *var* gene groups. Recent studies on *var* gene transcription and malaria severity in clinical isolates have, however, reached conflicting results [20–23]. This may be because severe malaria encompasses a variety of clinical syndromes (e.g. cerebral malaria, severe anaemia, respiratory distress, prostration) [2], and these syndromes may have different underlying pathogenic mechanisms [3]. Significant associations between *var* gene groups and clinical disease may be masked unless strictly defined clinical groups are assessed along with suitable control groups [24,25].

We aimed to investigate the hypothesis that parasite virulence and clinical disease severity in African children are associated with the transcription of distinct subsets of *P. falciparum var* genes. We examined *P. falciparum* isolates from patients with one of the commonest and most virulent forms of severe malaria, cerebral malaria (unrousable coma), compared to isolates from controls with equally high parasite burdens but non-virulent disease (hyperparasitaemia patients). Our data show that *P. falciparum* isolates from cerebral malaria patients are significantly more likely to transcribe *var* genes with DBLα1-like domains characteristic of Group A and B/A *var* genes than isolates from patients with non-severe hyperparasitaemia, which predominantly transcribe *var* genes with DBLα0-like domains.

## 2 Materials and methods

### 2.1 Sample collection and RNA extraction

The samples were collected as part of the Bandiagara Malaria Project case-control study of severe malaria that has been described in detail previously [26,27]. Blood samples were collected from children with malaria after informed consent from parents or guardians, and all protocols received institutional review board approval. Blood samples were suspended in glycerolyte after lymphocyte separation via density centrifugation and frozen to −70 °C. A blood spot from each isolate was made on 3 MM Whatman paper for genomic DNA extraction. Frozen samples were shipped to Edinburgh where they were thawed by standard methods and cultured for 8–24 h until the parasites were a mixture of late ring stage and early pigmented-trophozoite stage (morphologically equivalent to approximately 16–20 h post-invasion in a laboratory strain). This was done in order to study parasite stages in which the full length *var* mRNA is present [28], while avoiding the early ring stage, when some authors claim multiple non-specific *var* transcription occurs [29]. The cultured cells were solubilised in Trizol and stored at −70 °C. RNA was extracted as described [28].

### 2.2 DNA extraction and genotyping

DNA was extracted from blood spots on filter paper using chelex-100 extraction [30]. The minimum number of genotypes per isolate was estimated by genotyping PCR with primers to MSP1 and MSP2 [31].

### 2.3 cDNA preparation

RNA was treated for 30 min at room temperature with 1.5 units DNAase (Gibco) to remove any contaminating genomic (g) DNA. cDNA was prepared using Superscript First Strand Synthesis System (Invitrogen) with random hexamers according to manufacturers instructions.

### 2.4 RT-PCR

Reverse-transcriptase (RT)-PCR was used to amplify a region of 300–400 bp of the *var* DBLα domain using unbiased degenerate primers αAF′ and αBR [14,21]. These primers are capable of amplifying the entire *var* gene repertoire of 3D7 apart from the strain-transcending *var2CSA* gene implicated in malaria in pregnancy [32] and the type 3 *var* genes that are of unknown function. Amplification conditions were as described [14] with a hot start (95 °C, 5 min) followed by 35 cycles of 95 °C, 20 s; 42 °C, 20 s; 60 °C, 1 min. Samples without RT were used in all reactions to exclude gDNA contamination.

### 2.5 PCR for upstream regions

Upstream PCRs were carried out on gDNA using upstream primers UpsA (5′-TAT TYH ATK TAT TAY ATT TGT TGT A) UpsB (5′-GTT AGA ACA TTT AAA ATT ATA) and UpsC (5′-AVA GAW ATA TGR TAG ATA YAG), based on sequences from 3D7, and a gene-specific DBLα primer for each isolate. Amplification conditions were 35 cycles of 94 °C, 5 s; 46 °C, 15 s; 60 °C, 2 min. The upstream sequence of the predominant *var* gene from nine isolates was studied. The remaining upstream sequences were not studied due to lack of parasite material.

### 2.6 Cloning and sequencing

PCR and RT-PCR products were run on agarose gels and extracted (Gel Extraction Kit, QIAGEN) then cloned (TA cloning kit, Invitrogen), and used to transform One Shot TOP10F competent cells (Invitrogen). Transformed cells were grown overnight and individual white colonies were selected for culture. Plasmids were extracted from overnight cultures using a miniprep kit (QIAGEN), and were sequenced using BigDye terminator reaction mix (Applied Biosciences).

### 2.7 Sequence analysis

Sequences were analysed using Lasergene software (DNASTAR Inc). Contigs were created with a minimum percentage match of 95% to classify sequences for each isolate. Amino acid sequences were aligned using MUSCLE [33] and the alignment converted into a nucleotide alignment with Tranalign (http://bioweb.pasteur.fr/docs/EMBOSS/tranalign.html)

### 2.8 Phylogenetic network

A phylogenetic network was generated rather than a traditional phylogenetic tree because *var* genes are subject to recombination. The network allows visualisation of ambiguous and conflicting signals in the dataset due to recombination or other factors [34]. Splits computed from the data are represented as parallel edges rather than single branches and the network provides an implicit representation of evolutionary history [34]. The phylogenetic network was generated in SplitsTree 4.4 [34] using the Neighbor-Net distances transformation [35] and equal angle splits transformation [36].

## 3 Results

Twenty-six *P. falciparum* field isolates were obtained from a vaccine trial site in Mali with intense seasonal transmission of *P. falciparum* (up to 20–60 infected bites per person per month at the peak of the July–December transmission season [37]). Blood was obtained from children with cerebral malaria (unrousable coma with a Blantyre coma score ≤2 and no other detectable cause of coma), non-severe hyperparasitaemia (>500, 000 parasites per microlitre of blood but with no symptoms or signs of severe disease) or uncomplicated malaria (fever and *P. falciparum* parasitaemia with no evidence of severe malaria or hyperparasitaemia). Hyperparasitaemia is generally considered to be an indication of severe malaria using World Health Organisation criteria [38]. However, African children with hyperparasitaemia and no other symptoms or signs of severe disease have a very low mortality rate, and hyperparasitaemia in sub-Saharan Africa can thus be considered as a form of non-severe malaria [2,26].

There were no significant differences in patient age across the three clinical categories (Table 2). The cerebral malaria and hyperparasitaemia patients did not differ significantly in parasitaemia, but the uncomplicated malaria patients had significantly lower parasitaemias than the other two groups (Table 2). We therefore considered the hyperparasitaemia isolates to provide a better control group for the cerebral malaria isolates than the uncomplicated malaria isolates, because the major difference between the cerebral and hyperparasitaemia isolates is in parasite virulence and disease severity rather than parasite burden. Parasite rosette frequency was significantly associated with disease severity, as expected in isolates from Africa (Table 2) [15,16]. Multiple genotypes were present in most isolates (range 1–5), and there was no significant difference in the number of genotypes between the three clinical categories (Table 2).

**Fig. 1:**
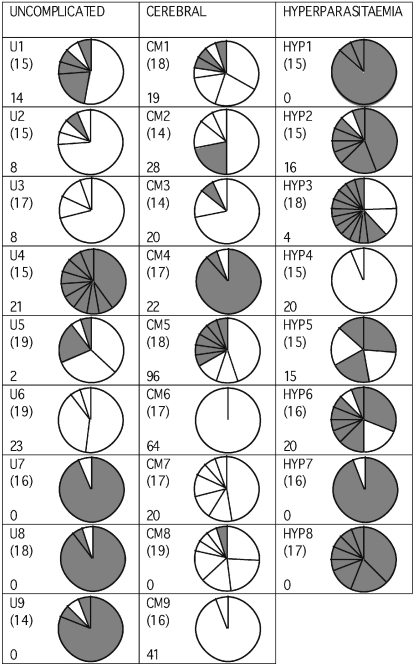
Frequencies of distinct DBLα tags in each isolate. The pie charts represent the relative number of different DBLα *var* gene sequences detected in each isolate by RT-PCR, cloning and analysis of 14–19 mini-prep clones per isolate. The number of sections in each pie represents the number of distinct DBLα tags detected, and the size of each section represents the relative frequency of each DBLα tag. Bracketed numbers indicate the exact number of mini-prep clones sequenced for each isolate. The sample name is in the top left corner of each box and the rosette frequency (percentage of mature infected erythrocytes binding two or more uninfected erythrocytes) in the bottom left corner. White segments represent DBLα1-like *var* genes (i.e. two conserved cysteine residues in the amplified DBLα tag) whereas grey segments represent DBLα0-like *var* genes (i.e. four conserved cysteine residues in the amplified DBLα tag).

The transcribed DBLα *var* gene sequences from each isolate were amplified by reverse transcriptase-PCR using unbiased degenerate primers [14]. The RT-PCR products were cloned and 14–19 recombinant plasmids with *var* gene inserts were sequenced per isolate. Identical sequences from the same isolate were defined as isolate sequence a, b, c etc. in decreasing order of abundance (GenBank accession numbers DQ367086–DQ367226). The number of distinct DBLα *var* gene sequences detected per isolate varied between 1 and 14, and almost every isolate showed a predominant gene (Fig. 1). Identical or almost identical sequences from different isolates were rare, indicating minimal overlap in *var* gene repertoires between isolates (see Supplementary material for further details). There was no significant difference in the number of distinct DBLα sequences per isolate detected in each clinical category (cerebral malaria: median 5, range 1–9; hyperparasitaemia: median 6.5, range 2–14; uncomplicated malaria: median 4, range 2–10, *P* = 0.72, Kruskall–Wallis test). Further experiments to validate the reproducibility of these data are shown in the Supplementary material (Fig. S1).

**Fig. 2:**
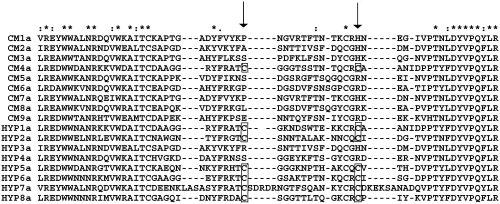
Alignment of DBLα domains. A MUSCLE alignment of the predominant gene transcribed by *P. falciparum* isolates from cerebral malaria and hyperparasitaemia patients. The second half of the expressed sequence tag is shown and the position of the residues that distinguish DBLα0-like domains (cysteines present) from DBLα1-like domains (cysteines missing) are arrowed, and the cysteine residues are boxed. The predominant gene in 8/9 isolates from cerebral malaria patients is of the DBLα1-like type, whereas the predominant gene in 6/8 hyperparasitaemia isolates is of the DBLα0-like type. This difference is significant by Fishers Exact test (*P* = 0.013). Symbols: (*) indicates conserved residues; (:) indicates conservative substitution; (·) indicates semi-conservative substitution.

There was a highly significant difference between clinical categories in the percentage of *var* genes amplified that were DBLα1-like. 80.1% of the *var* gene sequences amplified from the cerebral malaria isolates were DBLα1-like, compared to only 25.7% of the sequences from the hyperparasitaemia isolates and 40.5% of the sequences from the uncomplicated malaria patients (*P* < 0.001, Chi-square test, Fig. 1). If only the predominant gene from each isolate was examined, a marked difference remained between the cerebral malaria and hyperparasitaemia isolates (*P* = 0.013, Fishers Exact test, Fig. 2), but not between any other group (uncomplicated versus hyperparasitaemia, *P* = 0.294; uncomplicated versus cerebral, *P* = 0.131, Fishers Exact test). There was a significant positive correlation between the proportion of DBLα1-like sequences and the rosette frequency (Rho = 0.520, *P* = 0.009, Spearman Rank correlation).

**Fig. 3:**
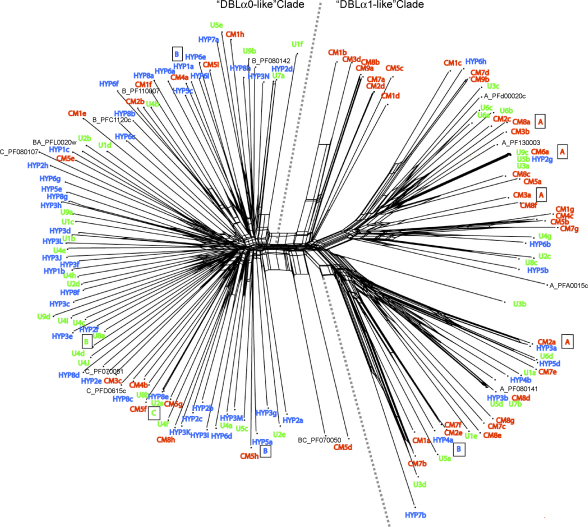
Phylogenetic network showing relationships between *var* gene DBLα sequence tags transcribed by *P. falciparum* isolates, generated using Neighbour-Net [35]. Sequences transcribed by isolates from African children with cerebral malaria (CM, red), hyperparasitaemia (HYP, blue), and uncomplicated malaria (U, green) are compared to a selection of *var* genes from the laboratory clone 3D7. For the 3D7 genes, the gene name is preceded by A, B, C, BA or BC to indicate the group to which the gene belongs. The sequences fall into two major clades, with the DBLα0-like sequences to the left of the dotted line and the DBLα1-like sequences to the right. For the predominant gene from nine of the Malian isolates, the *var* gene upstream region was determined by PCR and is indicated as a boxed letter.

The contrast between the *var* genes transcribed by the isolates from cerebral malaria patients compared to hyperparasitaemia patients was also demonstrated by mapping clinical categories across a phylogenetic network of DBLα sequence tags (Fig. 3). A phylogenetic network was used rather than a traditional phylogenetic tree because *var* genes are subject to recombination, a process that can generate conflicting signals in tree-building and can be a significant source of error [34]. For reference, a selection of DBLα sequence tags from the three major Groups A, B and C from the fully sequenced *P. falciparum* laboratory clone 3D7 [8] were included, plus four 3D7 genes with intermediate characteristics (B/A or B/C). In the phylogenetic network the sequences fell into two major clades (Fig. 3). All of the sequences to the left of the dotted line are DBLα0-like (i.e. they contain four cysteine residues in the expressed sequence tag), whereas all of the sequences to the right of the dotted line are DBLα1-like (containing two cysteine residues in the expressed sequence tag) (Fig. 3). A phylogenetic network containing the full repertoire of 3D7 *var* gene sequence tags plus the Malian sequences was also generated and gave the same separation into two distinct clades of DBLα0-like and DBLα1-like sequences (data not shown due to the complexity of the figure. This is available from the authors on request). Sequences from the cerebral malaria patients (red in Fig. 3) are significantly concentrated into the “DBLα1-like clade” (*χ*^2^ = 12.72 on 1 d.f., *P* < 0.001), while sequences from the hyperparasitaemia patients (blue) are significantly concentrated in a “DBLα0-like clade” (*χ*^2^ = 5.6 on 1 d.f., *P* < 0.025). Sequences from the uncomplicated malaria patients (green) showed no bias in distribution between the two clades (Fig. 3).

Further separation of the DBLα expressed sequence tags into the six sequence groups recently proposed by Bull et al. [21] did not reveal any further differences between the three disease categories (see Supplementary material, Fig. S2). Investigation of the *var* gene upstream regions for the predominant *var* gene from nine isolates showed that four field isolate *var* genes with DBLα0-like domains had UpsB or UpsC upstream regions, whereas four out of five *var* genes with DBLα1-like domains had UpsA-type upstream regions and the fifth (Hyp4A) had an UpsB sequence linked to a hybrid DBLα0/α1 domain and was therefore a Group B/A *var* gene (Fig. 3).

## 4 Discussion

The major finding of this study is that *P. falciparum* isolates from Malian children with cerebral malaria (a virulent form of disease) predominantly transcribe *var* genes with DBLα1-like domains that are characteristic of Group A or B/A *var* genes, whereas isolates from patients with hyperparasitaemia (a non-virulent form of disease) predominantly transcribe *var* genes with DBLα0-like domains. These results provide the first firm evidence from *P. falciparum* clinical isolates to support the hypothesis put forward previously that Group A *var* genes (which all have DBLα1 domains) could be implicated in the pathogenesis of severe malaria in African children [19].

Previous studies on *var* gene transcription in clinical isolates have given conflicting results. A role for *var* genes with DBLα1-like domains in the multi-organ failure type of severe malaria that occurs in adults in low malaria transmission settings was suggested by a study of 10 Brazilian isolates [20]. A study in Papua New Guinea, however, showed upregulation of Group B *var* genes in clinical disease (severe and uncomplicated) compared to asymptomatic infections [22]. The “severe disease” patients in the Papua New Guinea study had a variety of different clinical syndromes, and strictly defined cerebral malaria (Blantyre coma score ≤2) was not included. A recent study from Tanzania also investigated a mixed group of severe malaria patients and found increased abundance of both Groups A and B *var* genes in severe compared to uncomplicated and asymptomatic malaria patients [23]. Analysis of a sub-group of seven patients with neurological alterations (Blantyre coma score ≤3, therefore this group does not fulfill a strict definition of cerebral malaria) did suggest a trend towards increased abundance of Group A *var* genes, but this was not statistically significant [23]. A study of sequence diversity in Kenyan field isolates did not find any association between *var* gene group and disease manifestation, however only six isolates from assorted severe malaria patients were studied, only one of whom had cerebral malaria [21]. As pointed out previously, severe malaria is a heterogeneous group of syndromes that may have different underlying pathogenic mechanisms, therefore strict definitions of disease are essential in studies addressing malaria pathogenesis [24]. Appropriate controls groups with equivalent parasite burdens are also required [24,25], and the study reported here is the first to investigate *var* gene transcription using isolates from non-severe hyperparasitaemia patients as a control group rather than uncomplicated malaria patients with low parasite burdens. Although it remains possible that there are genuine geographical differences in the relationship between *var* gene transcription and disease severity, we suggest that the reason why a clear pattern emerged from the current study, even though the sample numbers are not large, is because a strictly defined severe disease syndrome (cerebral malaria) was investigated and a non-severe malaria control group with equivalent parasite burdens (hyperparasitaemia) was used.

We also investigated *var* gene transcription in parasite isolates from children with uncomplicated malaria and lower parasite burdens that are equivalent to the control groups used in previous studies [20–23]. Isolates from these patients showed a mixed pattern of *var* gene transcription with four out of nine isolates transcribing mostly DBLα1-like *var* genes, while five out of nine isolates transcribed mostly DBLα0-like var genes (Figs. 1 and 3 and Fig. S2). This is consistent with the idea that non-hyperparasitaemic uncomplicated malaria represents an earlier stage in the natural history or pathogenesis pathway of a malaria infection. At this stage, the host–parasite process has progressed beyond asymptomatic infection and produced symptoms, but it may yet diverge along several possible pathways ranging from spontaneous resolution to hyperparasitaemia, cerebral malaria, other severe syndromes, and death. Our data support the hypothesis that parasites expressing DBLα1-like *var* genes influence the direction of the disease process toward cerebral malaria, presumably increasing the predilection for parasites to sequester and cause microvascular obstruction in the brain. Other factors are likely to influence this process including host genotype [39], and it is possible that parasite isolates transcribing “cerebral malaria-type” DBα1-like *var* genes may fail to cause severe disease in some children because of host genetic polymorphisms that can modify parasite virulence phenotypes [40].

This study and previous similar investigations [20–23] use peripheral blood samples to examine *P. falciparum var* gene transcription profiles in clinical isolates. One unusual feature of malaria infection is that only immature ring stage parasites are found in the peripheral blood, whereas the mature blood stage parasites (pigmented trophozoites and schizonts) are sequestered in the microvasculature [3]. Therefore it is a major concern in studies of this type whether the parasites in the peripheral blood adequately represent the sequestered population. Ideally studies aimed at examining the relationship between *var* gene transcription and particular disease syndromes would examine sequestered parasites, however, these are only accessible in post-mortem samples making such studies technically and ethically difficult. Two recent studies from Malawi suggest that the dominant parasite genotypes of sequestered parasites are usually the same as those in the peripheral blood [41], and that parasite genotypes in cerebral malaria patients are homogeneously distributed throughout the body (i.e. distinct genotypes are not sequestered in the brain compared to elsewhere in the body or the peripheral blood) [42]. Although these studies do not shed light upon the question of whether the *var* gene profile of peripheral blood parasites reflects that of sequestered ones, they are consistent with the possibility that the peripheral blood population could adequately reflect the sequestered parasite mass. Any difference in *var* gene transcription between peripheral and sequestered parasite populations would be likely to confound a clear picture of the relationship between *var* genes and disease syndromes. The fact that such a clear pattern of *var* gene transcription in isolates from cerebral malaria patients compared to hyperparasitaemia patients did emerge from this study suggests that such a confounding effect is not a major problem.

Can the differing virulence-associations of the DBLα1-like *var* genes compared to the DBLα0-like *var* genes shown in this study be explained by the known functions of the different *var* gene groups? The only adhesion phenotype currently ascribed to the DBLα1 domain of some Group A or B/A *var* genes is binding to complement receptor 1 on uninfected erythrocytes to form rosettes ([17,18] and J.A. Rowe unpublished data). The field isolate data reported here along with two previous studies [21,22] supports a link between the transcription of *var* genes with DBLα1-like domains and high rosette frequencies. Rosetting is a parasite virulence-associated phenotype [15,16] that is thought to contribute to malaria pathogenesis by enhancing sequestration and microvascular obstruction [43]. The function of rosetting is unknown, but it may be an immune evasion mechanism that promotes parasite growth and survival leading to rapid high parasitaemia *in vivo* [44]. Whether *var* genes containing DBLα1-like domains mediate other binding phenotypes, in addition to rosetting, that promote virulence is unknown, and is an important question requiring further research.

*Var* genes encoding PfEMP1 variants with DBLα0 domains (i.e. Group B, C, B/C and some B/A *var* genes), on the other hand, have been shown to bind to the endothelial receptor CD36 [11], and this interaction is believed to lead to the sequestration of infected erythrocytes in non-vital tissues [45] (away from brain endothelium, which does not express CD36 [46]). Our finding that *var* genes with DBLα0-like domains are predominantly transcribed in non-virulent infections is consistent with this hypothesis. In addition, binding of infected erythrocytes to CD36 on dendritic cells leads to inhibition of dendritic cell maturation and impairment of host immune-responsiveness [47]. This immuno-modulatory effect of Group B and C *var* genes could contribute to the lower virulence of malaria in the hyperparasitaemia patients if, as has been suggested previously, immuno-pathology contributes to the pathogenesis of severe malaria [48]. Parasites from hyperparasitaemia patients have also been shown to have lower *in vitro* multiplication rates than parasites from patients in other disease categories [49], which could also contribute to the lower virulence of these infections.

In conclusion, we have shown that *var* genes with DBLα1-like domains (characteristic of Group A and some B/A *var* genes) are predominantly transcribed in *P. falciparum* isolates from Malian cerebral malaria patients, whereas *var* genes with DBLα0-like domains are predominantly transcribed in isolates from patients with a non-virulent form of disease (hyperparasitaemia). These findings suggest fundamental differences in the roles played by the different *var* genes groups in host–parasite interaction that could be related to the contrasting cytoadhesive and immuno-modulatory functions of the *var* gene groups. Further research is required to examine the disease-associations, functions and diversity of *var* gene subsets in different geographical areas, and we emphasise the importance of strict clinical definitions and appropriate control groups in future work.

## Figures and Tables

**Table 1:** Characteristics of *P. falciparum var* gene groups

*var* gene group	Upstream sequence	Position	Orientation (direction of transcription)	No. of genes in 3D7	No. of DBL domains	DBLα0/α1	No. of cysteines in amplified tag
A	UpsA	Subtelomeric	Telomeric	10	2–5	DBLα1	2
B/A	UpsB	Subtelomeric	Centromeric	4	4–7	DBLα1 or DBLα0^a^	2 or 4
B	UpsB	Subtelomeric	Centromeric	21	2–3	DBLα0	4
B/C	UpsB	Central	Telomeric	10	2–3	DBLα0	4
C	UpsC	Central	Telomeric	13	2–3	DBLα0	4

a Two of the 3D7 Group B/A *var* genes (PFL0020w and MAL6P1.4) have a DBLα0 domain and two (PF08_0140 and MAL6P1.316) have a hybrid DBLα0/α1 domain that is DBLα0-like at the N-terminus but DBLα1-like in the expressed sequence tag region (i.e. it has two rather than four conserved cysteine residues).

**Table 2:** Summary of patient and parasite isolate details

Malaria disease category	Age (months) (mean ± S.D.)	Parasitaemia (%) (mean ± S.D.)	Rosette frequency^a^ (%) (median:25th, 75th percentiles)	Genotypes (mean:range)
Uncomplicated	45.8 ± 28.0	3.3 ± 1.5	8.0:0, 15.8	2.4 (1–4)
Hyperparasitaemia	42.5 ± 16.9	11.9 ± 3.9	9.5:0, 18.0	2.6 (1–4)
Cerebral malaria	35.2 ± 22.0	12.3 ± 6.3	22.0:19.8, 46.8	2.2 (1–5)
*P* values	>0.45^b^	0.83 H:C^c^, <0.001 U:C/H	0.03^d^	>0.48^e^

a Percent of mature-stage infected erythrocytes that bind two or more uninfected erythrocytes.

b Uncomplicated: cerebral *P* = 0.46, uncomplicated: hyperparasitaemia *P* = 0.72, cerebral:hyperparasitaemia *P* = 0.68, Student's *t*-test.

c H: hyperparasitaemia; C: cerebral malaria; U: uncomplicated malaria. Student's *t*-test.

d Kruskal–Wallis test.

e Uncomplicated: cerebral *P* = 0.72, uncomplicated: hyperparasitaemia *P* = 0.72, cerebral:hyperparasitaemia *P* = 0.49, Student's *t*-test.
