# Supplementary material for: Differential var gene transcription in Plasmodium falciparum isolates from patients with cerebral malaria compared to hyperparasitaemia
Source: Mol Biochem Parasitol. 2006 Dec;150(2-4):211–8. doi: 10.1016/j.molbiopara.2006.08.005 (PMC2176080; doi:10.1016/j.molbiopara.2006.08.005)

Supplementary Material

Identical sequences

Identical or almost identical (>95% nucleotide identity) sequences from different isolates were rare. Only 12 of the 142 unique sequence tags were present in more than 1 isolate, the most common of which (sequence tag CM6a) was seen in 5 isolates. This sequence has no identical database match in Genbank. In addition, 1 sequence tag (CM8d) was common to 4 isolates and 1 sequence tag (CM1g) was common to 3 isolates. Both of these are homologues of var1CSA, a *var* gene subfamily known to be widely transcribed in field isolates (Winter et al Mol Biochem Parasitol. 2003; 127:179-91). 9 sequence tags were common to 2 isolates, and only one of these (CM9b) had an identical match in Genbank (sequence A4AFBR19, Accession number **AJ319697**). Members of the putative *var4* subfamily, described by Jensen et al [19], were not amplified in any of the isolates examined.

RT-PCR validation.

Additional experiments were carried out to assess the reliability and reproducibility of the RT-PCR method used to detect transcribed *var* genes. To ensure that the sequences obtained in Fig 1 accurately represented the cloned RT-PCR product, colony picking was repeated and 50 additional mini-prep clones were sequenced for two isolates (CM7 and CM9). The results based on 50 sequences were comparable to the original results obtained with 16-17 sequences (Fig S1). In particular, the percentage of sequence tags obtained that were DBLα1-like was not significantly different when 50 compared to 16-17 mini-prep clones were analysed (Chi-squared test, not significant), supporting the validity of analysis from 14-19 sequences in the isolates as a whole. As an additional control, the RT-PCR was repeated on three samples (CM7, CM9 and CM4) to determine if the assay was repeatable and whether the same predominant sequence was obtained from a second amplification (Fig S1). In all three cases

the predominant *var* gene was the same in the second amplification, although the minority sequences detected varied slightly in two out of the three isolates (Fig S1). The proportion of DBLα1-like sequences was not significantly different in the second amplification compared to the first (Chi-squared test, not significant).

Legend Fig S1.

RT-PCR validation. The pie charts in the left column represent the relative numbers of different DBLα sequence tags from the original 16-17 mini-prep clones sequenced for isolates CM9, CM7 and CM4. The middle column represents the resulting *var* gene sequence tag frequencies when a further 50 mini-prep clones were sequenced from the original cloned RT-PCR product for CM9 and CM7. The right column indicates the sequence tag frequencies when the RT-PCR and cloning was repeated from scratch for isolates CM9, CM7 and CM4. Each unique *var* gene sequence tag detected in each isolate is shown by a different colour. The isolate name and number of mini-prep clones sequenced (bracketed) are shown in the top left corner of each box and the percentage of sequences detected that were DBLα1-like is shown in the top right corner of each box.

Fig S1

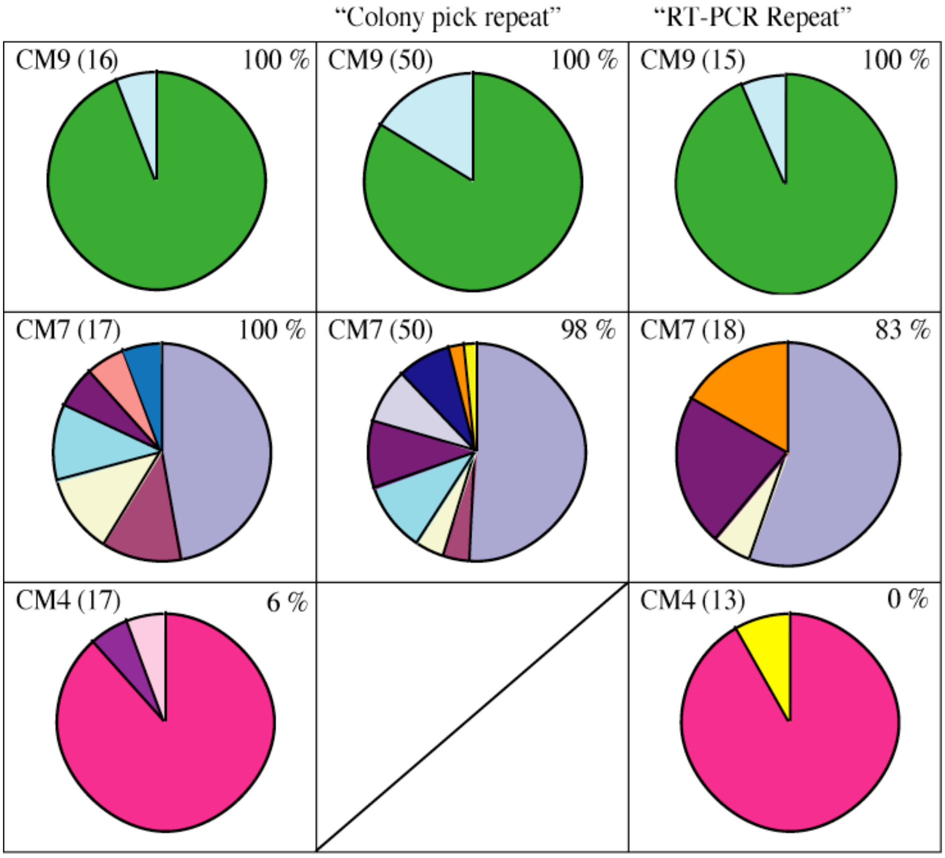
"Colony pick repeat"
"RT-PCR Repeat"
CM9 (16)
100 %
CM9 (50)
100 %
CM9 (15)
100 %
CM7 (17)
100 %
CM7 (50)
98 %
CM7 (18)
83 %
CM4 (17)
6 %
CM4 (13)
0 %

Analysis of *var* gene-virulence associations using sequence groups described by Bull et al [21].

We also analysed the data according the 6 sequence groups described by Bull *et al* in a recent paper on *var* gene DBLα diversity [21]. The *var* genes transcribed in the cerebral malaria isolates fall mainly into groups 1-3 (sub-types of DBLα1-like domains, classified on the basis of their sequence in positions of limited variability [21], whereas the *var* genes transcribed in the hyperparasitaemia isolates fall mainly into groups 4 and 5 (sub-types of DBLα0–like domain) (Fig S2). The group 6 described by Bull *et al* was not detected in any isolate. Within the DBLα1-like subgroups 1-3, the frequency of group 2 did not differ between cerebral malaria and hyperparasitaemia isolates (P= 0.70, Mann Whitney, Fig 4), whereas groups 1 and 3 were commoner in the cerebral malaria isolates (P= 0.0678 and P= 0.02, respectively, Mann Whitney, Fig S2). Rosette frequency was significantly correlated with proportion of group 1 genes (rho=0.394, P=0.049, Spearman Rank correlation) but not with any other group. This differs from the results of Bull *et al* who found that group 2 genes were associated with rosetting in 12 Kenyan field isolates [21].

There was some resolution of the Bull sequence groups in the phylogenetic network shown in Fig 3 (main manuscript). The upper cluster of sequences in the "DBLα1-like clade" (CM1b to U3b) corresponds to the Bull sequence groups 1 and 3, with the exception of CM7a/CM2d (a group 2 sequence that occurred in 2 different isolates). The lower cluster of sequences in the "DBLα1-like clade" of Fig 3 (CM2a to Hyp7b) corresponds to the Bull sequence group 2 with the exception of U5a (a group 3 sequence). These sequences (CM7a/CM2d and U5a) may represent point mutations in the positions of limited variability that define these groups, or recombinations between groups. There was no separation of Bull groups 4 and 5 within the "DBLα0-like clade".

Legend Fig S2. Proportions of transcribed *var* genes in each isolate falling into DBLα sequence groups 1-5 as described by Bull *et al* [21]. Most of the genes transcribed in the isolates from cerebral malaria patients are in groups 1-3, which are sub-types of Group A and B/A *var* genes with DBLα1-like domains classified on the basis of their sequence at positions of limited variability. The figure legend shows the number of cysteine residues and the sequence motifs in the expressed sequence tag that characterize each group according to Bull *et al* [21]. In contrast, the isolates from hyperparasitaemia patients mostly transcribe *var* genes in groups 4 and 5, which are sub-types of *var* genes containing DBLα0-like domains. The isolates from uncomplicated malaria patients show an intermediate pattern with 4 isolates transcribing mostly groups 1-3 (DBLα1-like) and 5 isolates transcribing mostly groups 4 and 5 (DBLα0-like). The proportions of sequences in each subgroup for all the isolates within each clinical category combined are shown at the right-hand side, and the differences between groups are statistically significant (P=0.0486, Kruskal Wallis test). The group 6 described by Bull *et al* was not detected in any isolate.

Fig S2

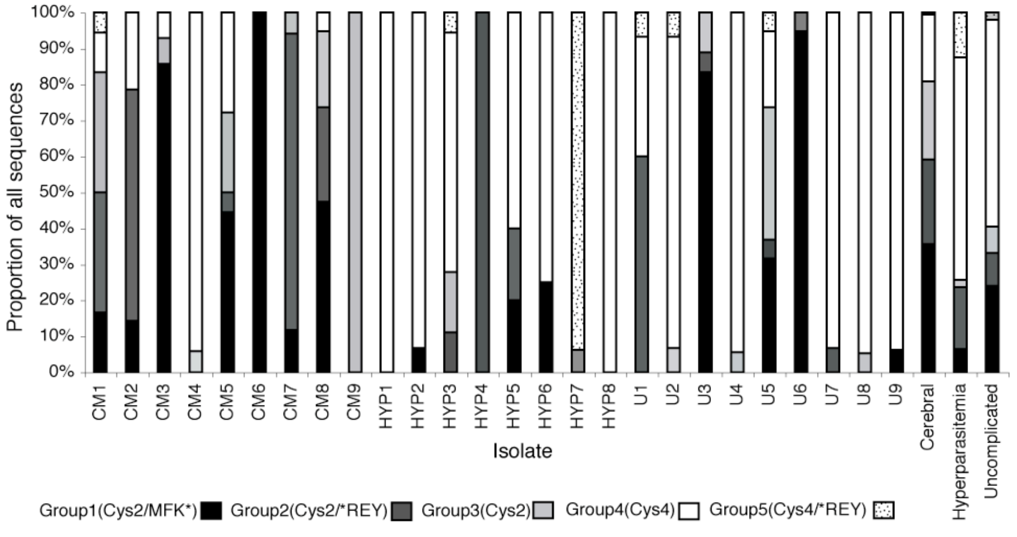

Supplement: Supplementary file 1 [file mmc1.pdf]
